# Improved resolution in the position of drought-related QTLs in a single mapping population of rice by meta-analysis

**DOI:** 10.1186/1471-2164-10-276

**Published:** 2009-06-22

**Authors:** Farkhanda S Khowaja, Gareth J Norton, Brigitte Courtois, Adam H Price

**Affiliations:** 1 Institute of Biological and Environmental Science, University of Aberdeen, Aberdeen, UK; 2 Biological Systems Department- UMR DAP TA A96/03, CIRAD, Montpellier, France; 3 Current address: Lethbridge Research Centre, Lethbridge, Canada

## Abstract

**Background:**

Meta-analysis of QTLs combines the results of several QTL detection studies and provides narrow confidence intervals for meta-QTLs, permitting easier positional candidate gene identification. It is usually applied to multiple mapping populations, but can be applied to one. Here, a meta-analysis of drought related QTLs in the Bala × Azucena mapping population compiles data from 13 experiments and 25 independent screens providing 1,650 individual QTLs separated into 5 trait categories; drought avoidance, plant height, plant biomass, leaf morphology and root traits. A heat map of the overlapping 1 LOD confidence intervals provides an overview of the distribution of QTLs. The programme BioMercator is then used to conduct a formal meta-analysis at example QTL clusters to illustrate the value of meta-analysis of QTLs in this population.

**Results:**

The heat map graphically illustrates the genetic complexity of drought related traits in rice. QTLs can be linked to their physical position on the rice genome using Additional file 1 provided. Formal meta-analysis on chromosome 1, where clusters of QTLs for all trait categories appear close, established that the *sd1 *semi-dwarfing gene coincided with a plant height meta-QTL, that the drought avoidance meta-QTL was not likely to be associated with this gene, and that this meta-QTL was not pleiotropic with close meta-QTLs for leaf morphology and root traits. On chromosome 5, evidence suggests that a drought avoidance meta-QTL was pleiotropic with leaf morphology and plant biomass meta-QTLs, but not with meta-QTLs for root traits and plant height 10 cM lower down. A region of dense root QTL activity graphically visible on chromosome 9 was dissected into three meta-QTLs within a space of 35 cM. The confidence intervals for meta-QTLs obtained ranged from 5.1 to 14.5 cM with an average of 9.4 cM, which is approximately 180 genes in rice.

**Conclusion:**

The meta-analysis is valuable in providing improved ability to dissect the complex genetic structure of traits, and distinguish between pleiotropy and close linkage. It also provides relatively small target regions for the identification of positional candidate genes.

## Background

In the past 20 years there has been a remarkable increase in the use of quantitative trait loci (QTL) mapping as a tool to uncover the genetic control of traits. Initially, a major motivation was a desire to locate useful genomic regions for use in marker-assisted selection (MAS) in breeding programmes. Increasingly, there is also a desire to identify the underlying genes responsible of agronomically or ecologically important QTLs. In addition to the great advances in genomics, such as whole genome sequencing, SNP discovery, transcriptomics and screening of large mutant libraries, progresses facilitating gene discovery have also been made in the area of biostatistics. An important example of this is the facilitation of meta-QTL analysis which uses data from independent experiments, each providing data on QTL position and confidence interval, to estimate the number of underlying "true" QTLs and obtain a more accurate estimate of meta-QTL positions. Meta-analysis allows distinctions between pleiotropy and close linkage to be made with greater confidence and provides a smaller region of the genome to explore for the underlying candidate genes.

Most commonly, meta-analysis of QTLs is applied to the compilation of data from independent mapping populations. Examples of this kind of analysis in plants include flowering time in maize [1] and wheat [2], nematode resistance in soybean [3], blast resistance in rice [4] and fibre development in cotton [5]. By definition, a meta-analysis is the combined analysis of multiple experiments and it can be used to provide a thorough analysis of one mapping population that has been used in multiple experiments [6].

The Bala × Azucena population of F_6 _recombinant inbred lines was developed in 1995 [7] and has been screened for root traits in controlled environments and for performance under drought in the field for the principle purpose of identifying QTLs for root traits in rice that might contribute to drought resistance. The initial motivation was to provide targets for marker-assisted breeding and, to that end, QTL information obtained from the population was used to identify four root growth QTLs that have subsequently been used in a MAS programme [8,9]. The population has also been used to study the stability of QTLs across environments [10]. A great deal of data has been generated on root growth and plant performance under drought. A compilation of this data has not been formally attempted until now. Doing so will aid attempts to identify positional candidate genes for the drought avoidance and root growth QTLs identified and help answer questions related to the pleiotropic effect of the underlying genes, which may prove very valuable in narrowing down positional candidate genes based on known function.

Here, a meta-analysis of root growth, drought avoidance and related plant growth QTLs is conducted. The value of analysis for distinguishing between pleiotropy and close linkage and in providing accurate locations for QTLs is explored using four exemplary loci. In order to facilitate wide use of these data, we provide a data set allowing the linking of the Bala × Azucena map to the rice sequence [see Additional file 1].

## Results

### Distribution of QTL across the genome

**Figure 1:**
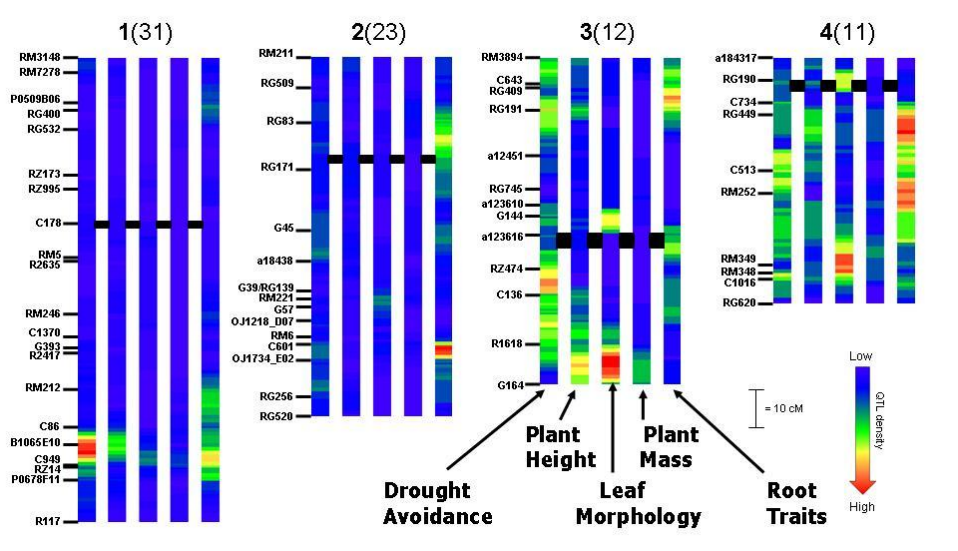
**Heat maps of chromosomes 1–6 of the Bala × Azucena mapping population showing QTLs classified in five trait categories**. The maximum QTL density for each chromosome is given in brackets after the chromosome number. Centromere locations are indicated by the large horizontal line.

**Figure 2:**
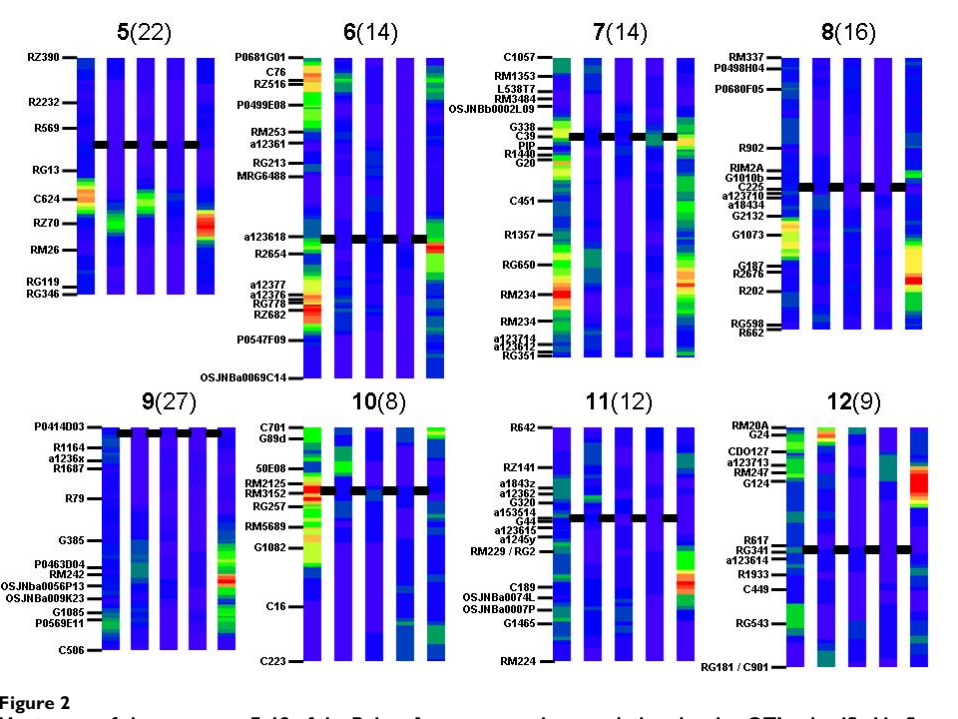
**Heat maps of chromosomes 7–12 of the Bala × Azucena mapping population showing QTLs classified in five trait categories**. See Figure 1 for description.

Figures 1 and 2 show the heat map generated from the one LOD confidence intervals of QTLs. Most markers used in the Bala × Azucena map can be aligned to the genetic and physical map of the International Rice Genome Sequencing Project sequence of rice [11] (the IRGSP map); this information is provided in Additional file 1. This will facilitate comparative genomics of the large data set analysed here with other studies in rice or other species.

Chromosome 1 had a region of high QTL density around B1065E10 for drought avoidance traits (maximum of 31 overlapping QTLs). The region was also active for plant height, leaf morphology, plant mass and root morphology. Root QTLs also concentrated slightly above RM212 and near the short arm telomere at P0509B06.

On chromosome 2, the highest density region was near C601 where 23 QTLs for root traits overlapped. Regions of high root QTL density were also present below G45 and below RG83, while there was a cluster of QTLs for leaf morphology at RM221 and clusters for drought avoidance above RG256, near C601 and near the AFLP marker a18438.

The maximum density of QTLs on chromosome 3 was only 12, most of which were leaf morphology QTLs near the long arm telomere (G164) of the chromosome. This region also carried a cluster of QTLs for plant height and plant mass, a cluster of leaf morphology QTLs and a small concentration of drought avoidance QTLs. Notable clusters for root QTLs were found at RG409, the AFLP a123616, and below C136. For drought QTLs two clusters near the short arm telomere, below RM3894 and at RG191, were visible, but most notable was a broad cluster from RZ474 to below R1618 on the long arm.

The most QTL dense region of chromosome 4 was near marker RG449 where 11 root QTLs overlapped. Root QTL activity appeared notable across most of the long arm of the chromosome. Leaf morphology QTL clusters were observed at RM349 (near where plant mass mapped) and RG190, while for plant height a cluster matching the root cluster below RG449 was apparent. Four clusters for drought avoidance were visible, near RG449, a broad area from above C513 to RM252, a region between RM252 and RM349 and a region at RM348.

On chromosome 5, each trait had only one region of QTL clustering. For root traits, 22 QTLs overlapped near marker RZ70, in the same place as a cluster of plant height QTLs. Notably higher (at marker C624) were clusters for drought avoidance, leaf morphology and plant mass.

Two regions of reasonably high QTL density (14) were detected on chromosome 6, root QTLs at R2654 near the centromere and drought avoidance on the long arm at RZ682. Both these trait categories had QTL clusters at the short arm telomere near C16, which also carries QTLs for plant height, while another drought cluster co-localized with the main root cluster near R2654. No notable activity for leaf morphology or plant mass was detected on chromosome 6.

Chromosomes 7 and 8 carried QTL clusters for root and drought traits. For chromosome 7, the most dense cluster was at RM234 where 14 QTLs for drought avoidance overlapped. Close by (between RG650 and RM324) was a dense cluster of root QTLs. A less dense cluster of root QTLs were also apparent at C39, and in a diffuse region around C451, while notable drought regions were C39, G20 and above RG650. Also noteworthy, however, was a cluster of plant mass QTLs between C39 and PIP, which, although from rather few individual QTLs, represented one of the strongest clusters of plant mass seen in this study. On chromosome 8, the densest QTL cluster was located near R2676 with 16 root QTLs. Marker RIM2A also was linked to a small root QTL cluster while there was a notable cluster of drought avoidance QTLs near G1073.

Chromosome 9 had a very dense region of 27 root QTLs just below marker RM242, but there might be two additional root QTL clusters, above G385 and below G1085. There was very little additional QTL activity on this chromosome, but some evidence of drought avoidance QTL at P0569E11 and of plant height at RM242.

Chromosome 10 had the least dense cluster, with 8 QTLs for drought overlapping at marker RM2125. Drought avoidance activity was widespread across the top half of the chromosome. Weak clusters of plant height (at 50E08) and root (at C701) QTLs were also apparent.

The only notable region of chromosome 11 was composed of 12 root QTLs at marker C189. On chromosome 12, 9 root QTLs clustered at marker G124, while plant height QTLs clustered at the short arm telomere, at G24. G24 was also near a weak cluster of drought avoidance QTLs, and there was another one between markers RM247 and RG543.

On the basis of the analysis described above, four regions were considered valuable examples for the purpose of addressing specific questions related to genetic architecture. Those questions were the precision of the QTLs position, the presence or absence of multiple QTLs on individual chromosomes and the distinction between pleiotropy and close linkage. These regions were; the dense drought avoidance QTL cluster at the bottom of chromosome 1 which might indicate pleiotropy with plant height and/or root traits; the QTL cluster on chromosome 5 which appeared to indicate one QTL for root and height traits, which did not overlap one for drought avoidance and leaf morphology; the drought avoidance QTL cluster at the top of chromosome 7 which appears in Figure 2 to be composed of two distinct meta-QTLs; and the root growth QTL cluster on chromosome 9, for which Figure 2 suggests there might be 3 separate meta-QTLs.

### Full meta-analysis of chromosome 1

**Figure 3:**
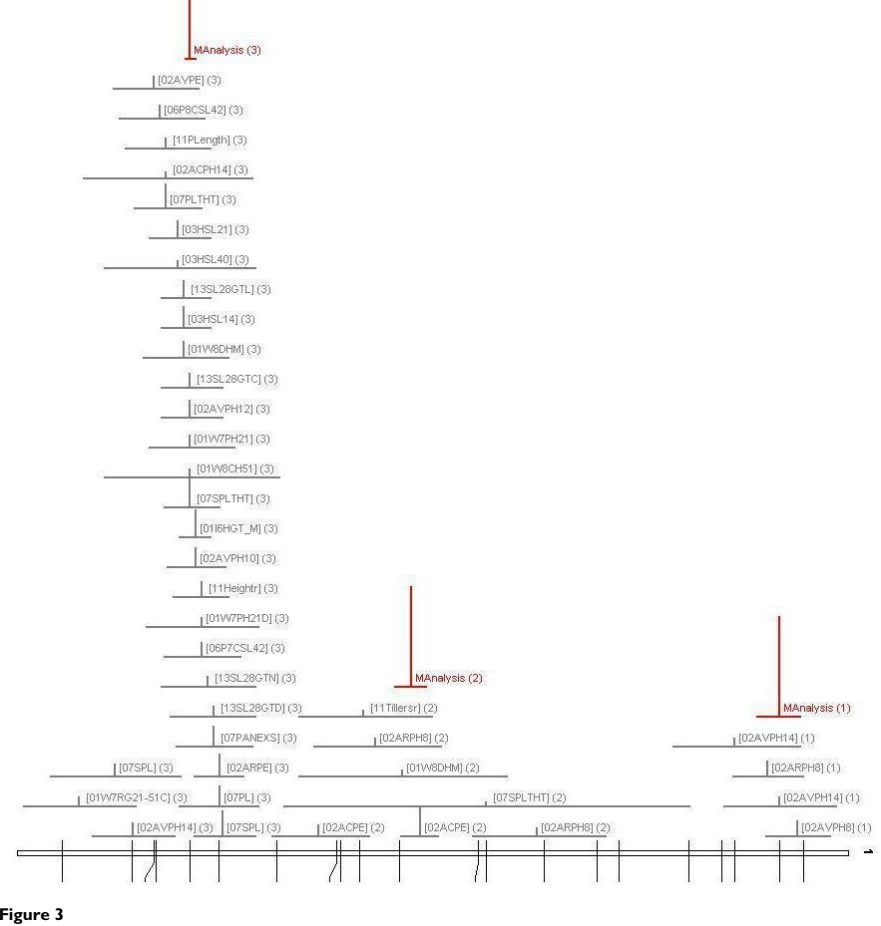
**Graphic of results of phase 1 meta-analysis of plant height QTLs on chromosome 1**. Analysis is performed by Biomercator and reveals three meta-QTLs.

In order to establish whether the cluster of drought avoidance QTLs at the bottom of chromosome 1 was pleiotropically related to plant height and root traits, a meta-analysis using BioMercator was conducted. In the first phase, 5 analyses were conducted, one for each trait category, with every QTL detected on that chromosome included. Figure 3 gives an example of the output, giving the best fit model for the 40 plant height QTLs which map to chromosome 1. It shows that 3 meta-QTLs were detected, the upper one near marker RM7278 with 4 individual QTLs, the middle one near marker RM246 with 7 individual QTLs and the lower one near marker B1065E10 with 29 individual QTLs.

A full summary of the first phase meta-QTL analysis of chromosome 1 is presented in the second and third column of Table 1. For drought avoidance, 71 QTLs were used and the analysis indicated that there were, most probably, four meta-QTLs on the chromosome, at 5.8 cM, 68.2 cM, 163.2 cM and 211.8 cM. For leaf morphology, the analysis indicated 4 meta-QTLs from 19 individual QTLs; for plant mass it was 2 meta-QTLs from 13 individual QTLs. For root QTLs, the model with the lowest AIC value was that with more than 4 QTLs. Therefore the individual QTLs were split into two groups and analysed separately. The split was located in a QTL-free region between 83 and 124 cM since there were 17 QTLs above this region and 58 below it. This revealed the presence of 6 meta-QTLs for roots on chromosome 1. Every trait category had a meta-QTL in the region of 205 to 220 cM (205 cM for height, 209 cM for plant mass, 212 cM for drought, 214 cM for leaf morphology and 220 cM for roots).

**Table 1:** Meta-QTL analysis of all QTLs on chromosome 1

Trait	Phase 1- all QTLs		Phase 2- one QTL per screen	
	
Category	Number of QTLs in meta-QTL	Meta-QTL Position (and 95% confidence interval)	Number of QTLs in meta-QTL	Meta-QTL position (and 95% confidence interval)
Drought Avoidance	8	5.8 (-1.1–12.7)		
	8	68.2 (62.2–74.1)		
	5	163.2 (156.9–169.4)		
	50	**211.8 (210.1–213.5)**	14	208.5 (205.9–211.2)


Plant Height	4	8.9 (1.6–16.2)		
	7	132.0 (126.5–137.4)		
	29	**205.3 (203.2–207.5)**	14	204.8 (201.9–207.8)


Leaf Morphology	12	**214.3 (210.9–217.6)**	7	215.2 (211.6–218.8)
	2	244.4 (233.1–255.7)		


Plant Mass	11	**208.6 (204.1–213.0)**	6	212.2 (206.6–217.7)
	2	243.3 (231.9–254.6)		


Roots	14	17.1 (11.4–22.8)		
	3	69.4 (59.5–79.4)		
	3	136.9 (129.2–144.6)		
	5	169.0 (160.5–177.5)		
	18	191.1 (187.3–194.8)		
	32	**219.6 (216.7–222.4)**	7	220.4 (215.9–224.8)

In this first phase of meta-analysis, no control was included over the number of individual QTLs entered into the analysis for each screen. It was considered possible that, for some screens, multiple QTLs could be detected for obviously pleiotropic traits and used in the meta-analysis (such as leaf rolling and leaf drying in the drought traits, or maximum root length and root mass at depth for the root traits) and that this would bias the mean position and confidence interval of the meta-QTL. Therefore it was thought prudent to include a conservative second phase analysis in which only one QTL for each independent screen of each experiment was used in the analysis. Where a choice of individual QTLs was possible, the QTL with the highest R^2 ^value was selected. The results are presented in the 5^th ^column of table 1. In most cases the mean position was close to that of phase 1, but the confidence interval was slightly larger because less individual QTLs were used. For plant mass, the 2^nd ^phase meta-QTL was 3 cM below the 1^st ^phase meta-QTL.

The plant height meta-QTL was located at 204.8 cM, only 0.2 cM from the marker B1065E10 at 205.0 cM. B1065E10 is an indel marker designed on BAC clone B1065E10 which contains the *sd1 *gene. This is the semi-dwarfing gene (Os01g66100; gibberellin 20-oxidase) [12] which has been partially sequenced in Bala, showing that Bala had the 383 loss-of-function deletion first identified in semi dwarf cultivars Dee-geo-woo-gen and IR8. Partial sequence of Azucena revealed it had the functional version of the gene. Since this gene is within 0.2 cM of the position of the plant height meta-QTL, it validates the approach being used here.

The *sd1 *gene, however, did not fall into the confidence intervals of the meta-QTLs for drought avoidance, leaf morphology, plant mass or root traits. This suggested that *sd1 *was not responsible for the cluster of non-height QTLs in this region. The confidence intervals of the leaf morphology and root meta-QTLs did not overlap that of the drought meta-QTL. Two clear and important conclusions from this analysis are apparent; *sd1 *is not a candidate gene for drought avoidance, root or leaf QTLs in this region; and root and leaf morphology QTLs here did not contribute to drought avoidance in this population.

### Full meta-analysis of chromosome 5

The phase 1 meta-analysis of chromosome 5 detected up to three meta-QTLs for each trait category (Table 2). It was notable, however, that every category had a meta-QTL between 73.4 and 88.3 cM where the majority of individual QTLs were placed. The second phase analysis concentrated only on these meta-QTLs. The meta-QTL position did not change by more than 1.2 cM from that of the first phase. Comparing the confidence intervals, it appeared that the meta-QTLs for drought avoidance overlapped substantially with those of leaf morphology and plant mass, but not with the plant height and root meta-QTL while plant height and root trait meta-QTLs were essentially overlapping. Leaf morphology and plant mass meta-QTL confidence intervals, which overlapped considerably, also overlapped at the extremes with the other traits. The simplest interpretation of these results was that there were 2 pleiotropic meta-QTL here, one at around 76–77 cM affecting drought avoidance, leaf morphology and plant mass, and another at 87 cM which affected plant height and root traits.

**Table 2:** Meta-QTL analysis of all QTLs on chromosome 5

Trait	Phase 1- all QTLs		Phase 2- one QTL per screen	
	
Category	Number of QTLs in meta-QTL	Meta-QTL Position (and 95% confidence interval)	Number of QTLs in meta-QTL	Meta-QTL Position (and 95% confidence interval)
Drought Avoidance	9	8.3 (3.8–12.8)		
	23	**73.4 (70.6–76.1)**	9	75.4 (71.0–79.8)
	4	113.8 (106.9–120.8)		

Plant Height	3	0.0 (-8.0–8.0)		
	15	**86.8 (81.8–91.8)**	11	86.4 (80.9–91.9)
	1	121.7 (109.4–134.0)		

Leaf Morphology	2	13.7 (1.5–25.9)		
	15	**76.8 (72.7–80.9)**	8	77.8 (72.9–82.6)

Plant Mass	7	**77.7 (71.7–83.7**)	5	76.2 (69.2–83.2)

Roots	7	17.87 (7.2–28.5)		
	25	**88.3 (85.0–91.5)**	10	86.8 (81.7–92.0)

### Full meta-analysis of the top of chromosome 7

As revealed in Figure 2, there is a region near the bottom of chromosome 7 at RM234 where several drought avoidance traits map. Higher up around the centromere, there is a region where there appears to be two close QTL clusters, one at C39 and the other at G20, markers that are 11.9 cM apart. A meta-analysis was conducted only for the higher clusters to test the hypothesis that there are indeed 2 meta-QTLs here. The best model using 19 individual QTLs indicated that there was only 1 meta-QTL, not 2, at position 50.5 cM (Table 3). The phase 2 analysis confirmed the conclusion and position, although the confidence interval, naturally, was larger. In both phases 1 and 2 of the analysis, therefore, the confidence interval was quite large (9.9 and 14.5 cM respectively). Nonetheless, the result suggested that, despite the fact that in the heat map there appeared to be two groups of drought avoidance QTLs whose 1 LOD confidence intervals did not overlap, as revealed by a region of blue (indicative of no confidence intervals) between the two clusters, the meta-analysis of Biomercator concluded they all belonged to one meta-QTL.

**Table 3:** Meta-QTL analysis of drought avoidance QTLs on the top of chromosome 7

Trait	Phase 1- all QTLs		Phase 2- one QTL per screen	
	
Category	Number of QTLs in meta-QTL	Meta-QTL Position (and 95% confidence interval)	Number of QTLs in meta-QTL	Meta-QTL Position (and 95% confidence interval)
Drought Avoidance	19	50.4 (45.5–55.4)	8	50.5 (43.2–57.7)

### Full meta-analysis of root traits on chromosome 9

Meta-analysis was conducted only on root traits on chromosome 9 to elucidate the number of meta-QTLs for the trait and get an accurate estimate of their position. A total of 58 individual QTLs were used in the first phase. Three meta-QTLs were identified all within a 50 cM region (Table 4), at 56.1 cM (some 3.4 cM above G385), 79.2 cM (0.7 cM below RM242) and 99.4 cM (0.9 cM above G1085).

**Table 4:** Meta-QTL analysis of root growth QTLs on chromosome 9

Trait	Phase 1- all QTLs		Phase 2- one QTL per screen	
	
Category	Number of QTLs in meta-QTL	Meta-QTL Position (and 95% confidence interval)	Number of QTLs in meta-QTL	Meta-QTL Position (and 95% confidence interval)
Roots	13	56.1 (52.1–60.2)	4	63.0 (57.2–68.9)
	29	79.2 (77.1–81.3)	8	80.1 (77.4–82.8)
	16	99.4 (94.9–104.0)	5	97.6 (91.5–103.7)

The upper meta-QTL contained individual QTLs in positions ranging from 0 to 61 cM and was dominated by root mass QTLs from the thin rhizotron experiment (experiment 06) (10 out of the 13 QTLs). From this experiment, four of the QTLs were QTLs for root mass in the 60–90 cm depth layer, two were QTL for root mass in the 90–120 cm depth layer, one was a QTL for total root weight and two were QTL for root/shoot ratio. Two QTLs for root penetration ratio from experiment 04 and for root/shoot ratio in experiment 03 (hydroponics) were also grouped in this meta-QTL. The middle meta-QTL contained individual QTLs for root thickness and mass from experiment 05 (soil-filled box 1), 06 (thin rhizotrons) and 13 (soil-filled boxes 2), but not for hydroponic root traits. The lower of these three meta-QTLs grouped mostly deep root trait QTLs (thickness of deep roots, deep root mass) and only QTLs from experiments 06 and 13.

Second phase analysis using only one QTL per screen gave very similar mean positions for the two lower meta-QTLs but the upper one shifted from 56.1 cM to 63.0 cM. This may be attributable to the fact that only a few individual QTLs were used to place this meta-QTL, and indeed, only 4 could be used in the second phase. This low number of individual QTLs introduces a large confidence interval for this meta-QTL and it must be considered that the position reported here is only approximate

## Discussion

### Identifying consensus QTLs by meta-QTL analysis

A great deal of QTL data from multiple experiments related to drought obtained using the Bala × Azucena mapping population has been compiled in a meta-analysis using two approaches. The first, illustrated in Figures 1 and 2, gives a heat map of 1 LOD ranges and clearly shows genomic regions rich in meta-QTLs for drought avoidance, shoot and root growth traits. The exercise has confirmed the presence of some regions previously identified as noteworthy. For example, a marker-assisted selection programme has introduced four genomic regions of Azucena into the Indian Upland variety Kallinga III with the aim of improving root growth [8]. These regions are all revealed as regions of meta-QTLs for roots in Figure 1 (near C601 on chromosome 2, RM234 on chromosome 7, RM242 on chromosome 9 and C89 on chromosome 11). While the chosen regions of chromosomes 2 and 9 stand out, it does appear that other regions might have been selected to be equally valuable as chromosomes 7 and 11, such as the regions around RZ14 on chromosome 1, RZ70 on chromosome 5, R2654 on chromosome 6, R2676 on chromosome 8 and G24 on chromosome 12. However, a limitation of the meta-analysis presented here is it does not account for the direction of the allelic effects. Thus for the meta-QTLs on chromosome 5, 8 and 12, the positive root alleles come from Bala, while for the QTLs on chromosome 6 the positive alleles come from both parents in different experiments. This indicates that a screening of the QTLs to be included in the meta-analysis is needed, and groups of QTLs having opposite direction effects should be constituted and treated separately. Previous reporting of individual experiments on field performance highlighted a number of regions carrying QTLs for drought avoidance traits (e.g. chromosome 1 near RZ14, chromosome 3 near C643, chromosome 5 near C624, chromosome 7 near C39 and chromosome 8 near G1073). The exercise here has identified two regions of interest for drought avoidance that had not appeared notable in individual experiments. These are the meta-QTLs on chromosome 6 (near RZ682) and 7 (RM234). This information is being used to compile lists of candidate genes in each region to identify gene standing out because they are present in multiple drought avoidance loci.

### Coincidence of drought avoidance QTLs with those of other traits

The first QTLs for physiological traits in rice [13] demonstrated co-localization of QTLs for field drought avoidance traits with QTLs for root traits measured in a controlled environment. Since then attempts have been made to confirm the relationship between roots and drought avoidance. The subject is not reviewed here, but the information presented in Figures 1 and 2 can be used to examine co-localisation of QTLs in this population in order to provide insight into physiological mechanisms underlying the drought avoidance QTLs. From Figures 1 and 2, 15 clusters of QTL for drought avoidance were considered notable, one on chromosomes 1, 4, 5, 8, 10 and 12, two on chromosome 7, three on chromosome 6 and four on chromosome 3. A clearly recognisable cluster of root QTLs is co-localizing with only five of these clusters (between RG409 and RG191 on chromosome 3, near both RZ516 and R2654 on chromosome 6, and near both PIP and RM234 on chromosome 7). For the QTLs around PIP and RM234 on chromosome 7, however, the direction of allelic effects of the drought avoidance QTLs and root QTLs are opposite; the Azucena allele improved drought avoidance but reduced root traits. Thus for only three drought meta-QTLs, can it be speculated that drought avoidance may be provided by root systems allowing increased access to water at depth.

Six of the drought avoidance QTLs appeared to be associated with plant height (at B1065E10 on chromosome 1, at RM3894, RG191 and above R1618 on chromosome 3, at RZ516 on chromosome 6, and between G24 and CDO127 on chromosome 12) while one (at C624 on chromosome 5) co-localized with QTLs for leaf morphology. In three cases, examination of allelic effects reveals that the taller plant alleles are associated with more severe drought symptoms. In the case of the QTLs on chromosome 3 near RG191 and that on chromosome 6 near RZ516, there is no consistent pattern of allelic effect, with positive alleles for both height and drought avoidance coming from both parents while in the case of chromosome 12, the allele for shorter plants is associated with more drought symptoms. On chromosome 5 the Azucena allele generally reduces symptoms of drought (although there are 3 QTLs with reverse effect) and it appears to reduce leaf area and weight, but increases chlorophyll content (per unit area).

Thus for a significant number of drought avoidance QTLs identified in the Bala × Azucena population (between RZ474 and C136 on chromosome 3, near C513 on chromosome 4, near RZ682 on chromosome 6, near PIP and RM234 on chromosome 7, near G1073 on chromosome 8, near RM3152 on chromosome 10 and at the top of on chromosome 12), the results do not indicate even possible theories about the underlying physiological mechanism. Interestingly, three of these regions match the eight clusters of drought responsive genes and previously identified QTLs for osmotic adjustment that have been recently reported [14] (27–32 Mb on chromosome 3, 22–27 Mb on chromosome 4 and 21–28 Mb on chromosome 8). Thus it is not unreasonable to hypothesise that osmotic adjustment, which has not been assessed in this population, may be the underlying mechanism in these meta-QTLs. Of the 22 high QTL density regions for clusters of root QTLs apparent in Figures 1 and 2, most did not appear to co-localize with plant height or leaf morphology high QTL density regions. There are only three exceptions, the strong cluster of root QTLs on chromosome 5 and the weaker clusters at the top of chromosome 6 and the upper QTL on chromosome 9. This firmly establishes that root traits are not strongly related to shoot traits in rice, unlike in some other cereals like barley [15].

These analyses imply that root QTLs do not contribute to drought avoidance in this population. However, as previously argued [16], caution is called for in reaching a firm conclusion on this issue because theoretically roots will only contribute to drought avoidance under certain circumstances (e.g. when the deep soil holds water and soil properties allow roots to exploit it). Aspects of soil properties have not been adequately addressed in studies on this population (or any other in rice) with the exception of experiment 09 [17].

### Accuracy of QTL position

It is still a matter of debate whether QTL analysis accurately reports QTL position or not. Price [18] showed that, for 20 QTLs which had either been cloned or accurately tagged, and for which the original QTL peak position was available, the distance from gene to QTL position averaged less than 2 cM, even for relatively small-effect genes. The conclusion that original QTL mapping can be accurate is confirmed to some extent here in the only case where the underlying gene responsible for a meta-QTL is know with any certainly; i.e. the *sd1 *gene for plant height on chromosome 1. In that case, 29 individual QTLs with LOD scores ranging from 2.6 to 15.0, R^2 ^values of 6.6% to 28% and peak positions from 194 to 242 cM were combined into one meta-QTL right on top of the gene. There is no relationship between the LOD score or R^2 ^value and the distance from the individual QTL peak position and the meta-QTL position, suggesting that the strength of the QTL is not related to accuracy which contradicts theory since heritability and precision are theoretically linked [19]. However, only 38% of individual QTLs were within 2 cM of the gene and 55% within 5 cM, suggesting that multiple QTL experiments do need to be conducted if accurate positions of relatively small QTLs are to be obtained.

### Quantifying the number of QTLs in a genomic region

One use of meta-QTL analysis is to identify if a genomic region that displays QTL activity really represents one, two or more underlying loci. This information is crucial if accurate QTL positions are to be obtained for marker assisted selection or listing positional candidate genes. Here, two examples of the value of meta-analysis for this goal are given. In the case of the top of chromosome 7 where Figure 2 suggests there are two clusters of high QTL density for drought avoidance, the meta-analysis reveals there is most likely only one. In the case of chromosome 9 where Figure 2 suggests the presence of two or three regions of high QTL density for root traits, the meta-analysis confirms that three are present, greatly increasing the accuracy with which each can be located on the genome.

### Distinguishing between pleiotropy and linkage

Another crucial question in QTL analysis is the degree to which the loci display pleiotropy as distinct from an apparent pleiotropy that results from close linkage. Strictly, the analysis can only reject pleiotropy in favour of close linkage and not the other way around since linkage between adjacent genes is not likely to be broken. Here we tested two cases where the distinction between pleiotropy and close linkage was not obvious. On chromosome 5 where meta-QTLs for all 5 categories of traits are present within a 15 cM region, Figure 1 suggests that both pleiotropy and close linkage may be acting since there appears to be meta-QTL for drought avoidance and leaf morphology in the same place, and meta-QTLs for plant height, mass and root traits lower down. The full meta-analysis confirms the presence of close linkage since it indicates no overlap in the confidence intervals of drought with root or plant height meta-QTLs. The situation for the plant mass and leaf traits is not conclusively elucidated because their meta-QTL positions are between those of the drought avoidance and both the root/height meta-QTLs, and because the confidence intervals of those meta-QTLs are quite large due to the lower number of individual QTLs used.

In the case of chromosome 1, Figure 1 does not help in deciding if the presence of meta-QTLs for all traits near RZ14 is due to close linkage or possible pleiotropy. The full meta-analysis, however, indicates strong evidence for close linkage in relation to a meta-QTL for drought avoidance above a meta-QTL for root traits. The confidence interval of the drought avoidance meta-QTL overlaps that of the plant height and plant mass QTLs but not that for leaf morphology. This suggests that if the drought avoidance QTL is pleiotropically related to shoot growth traits, it can be only with plant height and not with leaf length. Indeed, the plant height and leaf morphology traits do not appear to be pleiotropic, suggesting that the semi-dwarfing gene *sd1 *is not the only gene in this region affecting shoot growth traits.

## Conclusion

Other than generally advancing our understanding of the genetic architecture of QTL distribution, the main practical value of meta-QTL analysis is in the proposed ability to provide an accurate estimate of QTL position which will be useful in marker assisted selection and candidate gene identification. For marker assisted selection, the accurate location can be used to design ideal markers that reduce the likelihood of target gene loss due to distance between marker and gene. The greater resolution afforded for distinguishing between pleiotropy and close linkage is also valuable because it will allow breeders to target QTLs that have been neglected in the passed because they were thought to be pleiotropic with QTLs for undesirable traits but are now revealed to be distinct and separable by recombination. For example, the region of chromosome 1 described here which affects drought avoidance and plant height should be separable from the region that affects leaf length.

For the identification of positional candidate genes underlying the QTL, the meta-analysis should prove highly valuable. The confidence intervals obtained in this exercise vary from 5.1 to 14.5 cM with an average of 9.4 cM which compares favourably with the average 1 LOD interval for individual QTLs in this population of approximately 15 cM using composite interval mapping. While 9.4 cM is still on average about 180 genes in rice (based on 38,000 genes spread evenly over 2,000 cM), it is an amount that is becoming tractable as genomic resources and knowledge of gene function improves.

## Methods

### Phenotypic data

Here we use the term "experiment" to describe a specifically designed study using the Bala × Azucena mapping population and involving a number (between 1 and 8) of independent screens where each screen may or may not have replication. Table 5 summarises the 13 experiments on drought or root traits from which the phenotypic data have been compiled. Each experiment has been given a number based on chronology, thus the experiment number 01 was the first conducted and 13 the most recent.

**Table 5:** Experiments used for the meta-analysis of QTLs in the Bala × Azucena population.

Experiment number/description	Traits measured	Population size	No of Screens	Number of replicates of population per screen	Reference
01/Drought 1 IRRI^# ^and WARDA^# ^at vegetative stage	Drought avoidance, plant growth, leaf dimensions, Δ^13^C	110–176	4	2	[16,20]

02/Drought 1b WARDA at vegetative and reproductive stage	Drought avoidance	114	3	1	Not published

03/Hydroponics	Root traits	204	1	1	Not published

04/Root penetration	Root penetration	104	1	2	[7]

05/Soil-filled box 1	Root traits, gas exchange	170	2	1	[24]

06/Thin rhizotrons	Root traits, plant growth, drought avoidance	115–133	4	1	[25]

07/Drought 2 IRRI at reproductive stage	Drought avoidance, plant growth and yield	96	2	4	[21]

09/Field root screen, WARDA	Drought avoidance and root density	114	2	2 and 3	[17]

10/Leaf traits	Stomatal conductance, leaf rolling and leaf dimensions	176	1	3	[26]

11/Drought 3 Tamil Nadu at reproductive stage	Drought avoidance, plant growth and yield	177	1	2	[22]

13/Soil-filled boxes 2	Root traits, plant growth, drought avoidance	168	4	2	[9]

IRRI = International Rice Research Institute, WARDA = West Africa Rice Development Association.

### Field experiments with drought stress

The population was grown in the field at the International Rice Research Institute (IRRI) in 1996 and 1998, and at the West Africa Rice Development Association (WARDA) in 1997 and 1998 to asses drought avoidance traits [20]. Each of these 4 individual dry season screens had 2 replications of the mapping population which were stressed by withholding irrigation during the vegetative stage of growth. Traits measured include leaf rolling, leaf drying and relative water content as reported in [20], traits related to leaf area, leaf weight and carbon isotope discrimination as reported in [16], plus biomass and tiller number, canopy temperature (by infrared thermometer), leaf chlorophyll content (SPAD meter) and plant height traits that have not been previously reported. In addition, during the WARDA 1997 screen, an additional set of 114 RILs were screened in a neighbouring field using an augmented design without replication of the RILs for similar traits when exposed to drought during the vegetative and reproductive phase of growth (35 days of stress starting at 16 or 46 days after sowing respectively). This data has not been previously reported.

A subset of 96 RILs was tested again at IRRI in two years and two water treatments with two replications, with drought avoidance, plant growth and yield being measured [21]. A drought experiment with a single screen of 177 RILs with two replicates was conducted in Coimbatore, Tamil Nadu, India in which leaf rolling, leaf drying canopy temperature, chlorophyll content (SPAD) and yield were measured [22].

In 2001, an experiment was conducted at WARDA with two screens of 2 or 3 replicates in fields with contrasting soil physical properties by ceasing irrigation for 21 days starting at 35 days after sowing. Root density at 30 cm depth was assessed at 70 days. Data on leaf rolling, leaf drying, relative water content and height were collected during the experiment. These data are reported in [17] and have been submitted for publication.

### Controlled environment experiments

All but one of the 205 lines of the mapping population were screened for root growth in a hydroponics experiment under conditions essentially identical to those described in [23] except that it was a growth chamber with 12 hr day at 200 μMol s^-1 ^m^-2 ^PAR and temperatures 25/28°C day/night (unpublished). Maximum root length was measured weekly until harvest at 40 days when root thickness, volume and dry weight were measured. The experiment was augmented with 16 replicates of each parental genotype, and while most RIL lines were unreplicated (present only once), 18 randomly chosen lines were present as duplicates in order to occupy the available space. In these replicated few where broad sense heritability can be calculated, it was high (e.g. 82% for max root length and 63% for root volume) highlighting the robustness of this data.

Two screens were conducted in a soil-filled box in a greenhouse with 170 RILs, one box being well watered and the other receiving drought as water was not applied after sowing. Root traits and gas exchange were measured after 4 weeks [24]. A wax layer was used to assess root penetration ability in 104 RILs, replicated over two runs providing data on the number and proportion of roots which got through an 80% wax layer after 24 days [7]. Between 115 and 133 lines were screened under greenhouse conditions in two years in two water treatments in thin soil-filled rhizotrons. Root traits, plant growth and drought avoidance traits (leaf rolling and relative water content) were measured up to 56 days [25]. Using large soil-filled boxes in a high light growth room (900 μmol s^-1 ^m^-2 ^PAR), 168 RILs were screened for root traits under four treatments, a well watered control or under low light (450 μmol s^-1 ^m^-2 ^PAR), low water or low nitrogen [10]. Root and shoot traits were measured at 28 days after sowing. An experiment on the leaf rolling and stomatal behaviour of excised leaves was conducted on 176 RILs in a growth room under approximate 450 μmol s^-1 ^m^-2 ^PAR [26].

### QTL analysis

All data was reanalysed using the most recent map which contains 164 markers covering 1832.8 cM on 12 linkage groups. QTL analysis was achieved by composite interval mapping conducted with QTLCartographer version 1.15 (C.J. Basten, B.S. Weir, and Z.B. Zeng, Department of Statistics, North Carolina State University) with model 6 using the program Srmapqtl set for "forward stepwise regression with background elimination" to identify significant background markers, and having a window size of 10 cM. Only QTLs with a LOD score of 2.5 or above were used for the analysis below. Normally, permutation analysis of trait data in this population to determine the 5% genome-wide threshold of significance gives LOD values of 3.0–3.3. We consider it important to include putative QTLs with LOD between 3.0 and 2.5 because these may be real QTLs despite being below the stringent genome-wide threshold, but acknowledge that 2.5 is a somewhat arbitrary threshold.

All trait data was divided into 5 broad categories depending on the type of trait. These were traits related to **drought avoidance **(leaf rolling, leaf drying, relative water content, canopy temperature, Δ^13^C; total of 115 traits giving 527 QTLs), **plant height **(including seedling, vegetative and reproductive stage plants where, in the latter it is panicle height that is measured; total of 61 traits giving 313 QTLs), **plant mass **(biomass; total of 21 traits giving 104 QTLs), **leaf morphology traits **(leaf length, width, specific leaf area, chlorophyl content (SPAD); total of 37 traits giving 201 QTLs) and **root traits **(maximum root length, root thickness, root mass, root mass at depth; total of 121 traits giving 505 QTLs).

### Aligning Bala × Azucena map to genome sequence

The physical coordinates of the RFLP and microsatellite markers used in the Bala × Azucena map on the rice sequence were obtained from Gramene . When Gramene did not report the sequence coordinates, a BLAST search of RFLP probe or SSR primer sequence was conducted at NCBI  and the coordinates of the homologous rice chromosome used.

### Heat map of 1 LOD ranges

The production of colour-coded intensity maps was performed in Visual Basic 6 as described in [27]. For each of the 5 trait categories, and for each chromosome, the map positions delimiting the 1 LOD range around each QTL peak was recorded. Then, QTL density at each location on the map was determined by integrating the overlapping 1 LOD ranges. The maximum intensity for each chromosome was calculated by using the maximum number of overlapping QTLs on each chromosome (from any of the trait categories). This maximum value then allowed the colour scale to be adjusted so as to produce intensity maps with the best possible contrast in colours, although this means that the colour intensity range is different for each chromosome.

### Meta-analysis with BioMercator

For 4 genomic regions which were identified in the heat map and for which specific questions were posed about the nature of QTL distribution, the relevant QTL with LOD ≥ 2.5 were used for a meta-analysis using BioMercator [28]. This programme has been designed to conduct two phases of meta-analysis of QTLs, the first being the merging of different marker maps and the second being the statistical analysis of meta-QTL position. Hence most applications to date have been merging data from independent experiments on different mapping populations and the root QTL data used here is being exploited in that way to gain insight into root growth QTLs across rice germplasm [29]. However, according to [6] "Biomercator can be applied to experiments where QTL are detected for different traits on the same population grown in different environments (Goffinet and Gerber 2000)". That is the application presented here.

This programme takes as input the peak position, the R^2 ^value, the population size and the left and right position of the 1 LOD interval. Using the data from these multiple individual QTLs it calculates a test statistic (a modified Akaike's criterion) for a model in which there is one, two, three, four or more than four meta-QTLs on the chromosome. The model with the lowest test statistic is the most probable model. In each model, a confidence interval is calculated for each meta-QTL. The analysis was undertaken in two phases, the first to determine the number of QTLs present on the chromosome, and the second to determine the most accurate location and confidence interval for the meta-QTL. In the first phase, all QTLs for each trait category and chromosomes were analysed. Then, in the second phase, concentrating only on individual QTL that are assigned to one focal meta-QTL, only one individual QTL is taken from each individual screen to avoid weighting those screens in which multiple traits map to the same place Thus, in the second soil-filled box experiment (experiment 13 [10]) where 4 screens were conducted (each with 2 replicates), no more than one QTL was taken from each screen, so no more than 4 QTLs could come from that experiment. The selection of QTL was based on the largest R^2 ^value. In addition, in the second phase of the analysis individual QTLs from unreplicated and unpublished experiments (experiments 02, 03 and 05 in table 5) were not included.

## Authors' contributions

FSK produced Additional file 1 and conducted an initial compilation of QTLs in the population. GJN formatted data for and produced the heat map. FSK and GJN together conducted the Biomercator analysis. BC advised on the compilation of QTLs and analysis for formal meta-analysis. AP provided the funds, directed the data compilation and presentation and wrote the first draft of the manuscript. All contributed to revisions of manuscript.

## Supplementary Material

Additional file 1 **Alignment of markers on the Bala × Azucena map of September 2007 to genetic and physical position on the map-base sequence of rice**. This files shows the position of markers used in the Bala × Azucena map in (Kosambi) cM, the position on the International Rice Genome Sequencing Project map where alignment is possible, the gene model to which the marker belongs where applicable, the position of the marker on the Nipponbare sequence if available and, if not available the position on the Nipponbare sequence of the PACor BAC clone which that marker hybridizes if that is available. The column for alternative names are abbreviated names of some markers used by the authors in previous publications. Click here for file
